# MCTP1 and MCTP2 promote ER–PM contact sites and regulate PI4P homeostasis and cell migration

**DOI:** 10.1091/mbc.E25-09-0445

**Published:** 2026-04-06

**Authors:** Suganthan Amirthagunanathan, Maxime Boutry, Vasudeva Tati, Dibyayan Maity, Caroline Chapman, Madhura R. Pandkar, Brian Raught, Peter K. Kim, Amit S. Joshi

**Affiliations:** ^1^Department of Biochemistry and Cell and Molecular Biology, University of Tennessee at Knoxville, Knoxville, TN 37916; ^2^Cell and Systems Biology Program, Hospital for Sick Children, Toronto, ON M5G0A4, Canada; ^3^Department of Medical Biophysics, University of Toronto, Toronto, ON M5G1L7, Canada; ^4^Princess Margaret Cancer Centre, University Health Network, Toronto, ON M5G1L7, Canada; ^5^Department of Biochemistry, University of Toronto, ON M5G0A4, Canada; Northwestern University

## Abstract

Endoplasmic reticulum–plasma membrane (ER–PM) contact sites play important roles in maintaining lipid homeostasis at the plasma membrane (PM), cellular calcium homeostasis, and cell signaling. Here, we show that MCTP1 and MCTP2 are at ER subdomains that form membrane contact sites (MCS) with multiple organelles using a proximity labeling assay. MCTPs are three C2 domain-containing transmembrane proteins. We show that upon overexpression, MCTPs promote ER–PM contact sites in a C2 domain-dependent manner. MCTP C2 domains bind to PI(4)P and PI(4,5)P_2_, phosphoinositides that are enriched in the PM. Furthermore, we show that deletion of MCTP1 or MCTP2 increases PI(4)P levels in the PM and promotes cell migration. Thus, our study identifies MCTPs as multiple ER-organelle contact site proteins and establishes its role at ER–PM contact sites in regulating lipid homeostasis and cell migration.

## Abstract (executive summary)

- MCTP1 and MCTP2 are at multiple ER-organelle membrane contact sites, and MCTP2 promotes ER–PM contacts through C2 domains that bind phospholipids such as PI4P and PI45P.
- Depletion of MCTP1 and MCTP2 leads to an increase in PM PI4P levels and cell migration.

## INTRODUCTION

Membrane contact sites (MCS) are sites of close apposition of the membranes of two organelles. MCS plays a major role in intracellular signaling, lipid homeostasis, organelle trafficking, and many other cellular processes (English and Voeltz, 2013; Helle *et al.*, 2013; Prinz, 2014). The endoplasmic reticulum (ER) makes dynamic as well as stable contact with different organelles in the cell (Wu *et al.*, 2018). ER–PM were first reported in close proximity in muscle cells in the 1950s (Porter and Palade, 1957). In ER–PM contact sites, membranes come within less than 30 nm and span 100 to 400 nm (Wu *et al.*, 2007; Chang *et al.*, 2017). ER–PM contact sites play critical roles in the regulation of calcium dynamics and plasma membrane (PM) lipid homeostasis (Gallo *et al.*, 2016; Saheki and De Camilli, 2017; Stefan, 2020; Li *et al.*, 2021).

ER–PM contacts sites are enriched in unique proteins that enable them to carry out these functions. One group of proteins is tether proteins, which establish ER–PM contact sites by bringing both membranes together (Gallo *et al.*, 2016). Tether proteins share characteristics such as an ER-anchored domain, a distal domain that binds either unique lipids or membrane proteins in the PM, and a large enough span to bridge the ER and PM (Henne *et al.*, 2015). In yeast, a proteomic approach-based study for ER–PM tethers identified tricalbins (Tcb1/2/3), Scs2, Scs22, and Ist2 as major tethers (Manford *et al.*, 2012). Mammalian homologues of these proteins are extended synaptotagmins (E-SYT1/2/3), vesicle-associated membrane protein (VAMP)–associated proteins (VAPA and VAPB), and TMEM16, respectively (Li *et al.*, 2021). Additionally, junctophilins and Sec22b have also been shown to act as tethers in PM–ER contact sites (Li *et al.*, 2021). Most of the proteins at the ER–PM contact sites interact with the PM through their lipid-binding domains. ESYTs bind PI(4,5)P_2_ through the C2 domains (Saheki *et al.*, 2016), OSBPL3, 5, and 8 bind PI(4)P and PI(4,5)P_2_ through pleckstrin homology domain (Chung *et al.*, 2015; Ghai *et al.*, 2017; Gulyás *et al.*, 2020), Nir2/3 binds to PA through the LNS2 domain (Kim *et al.*, 2013), and GRAMD1 binds cholesterol via the GRAM domain (Naito *et al.*, 2019).

ER–PM contact sites are a major lipid transfer protein-mediated lipid exchange site, especially regulating the phosphatidylinositol (PI) cycle (Gallo *et al.*, 2016; Saheki and De Camilli, 2017; Li *et al.*, 2021). ESYT1 exchanges DAG from PM to the ER following PI(4,5)P_2_ hydrolysis due to PLC activation. This exchange is Ca^2+^-dependent and mediated by the SMP domains (Saheki *et al.*, 2016; Bian *et al.*, 2018). Another SMP domain containing ER–PM contact site protein, TMEM24, transfers PI from ER to PM in response to PI(4,5)P_2_-mediated signaling (Lees *et al.*, 2017). OSBPLs are a group of ER-contact site proteins that also play a role in lipid transport. OSBPL5 and 8 counter-transport PI(4)P and PS between the ER and PM through their OSBP-related domain (Chung *et al.*, 2015). Another study indicates potential exchange of PI(4,5)P_2_ and PS by OSBPL5 and 8 (Ghai *et al.*, 2017). OSBPL3 has been shown to play a role in the transport of PI(4)P, PA, and cholesterol at ER–PM contact sites (Gulyás *et al.*, 2020). Nir proteins regulate the PI cycle, as Nir2 and 3 have been shown to exchange PA for PI at the ER–PM MCS (Kim *et al.*, 2013; 2015; Chang and Liou, 2015). GRAMD1 plays an important role in maintaining PM cholesterol homeostasis using StART domain (Sandhu *et al.*, 2018; Naito *et al.*, 2019).

ER–PM contact sites also play a critical role in regulating Ca^2+^ dynamics through store-operated calcium entry (Saheki and De Camilli, 2017; Balla, 2018; Li *et al.*, 2021). When ER calcium is depleted, STIM1 oligomerizes and interacts with PI(4,5)P_2_ in the PM. This relocalization of STIM allows it to interact with ORAI through its CAD/SOAR domain. This step allows activation of ORAI channel and influx of Ca^2+^ into cells (Liou *et al.*, 2005; 2007; Roos *et al.*, 2005; Feske *et al.*, 2006; Luik *et al.*, 2006; Prakriya *et al.*, 2006; Xu *et al.*, 2006).

Multiple C2 domain-containing transmembrane proteins (MCTPs) are ER-resident C2 domain-containing proteins (Shin *et al.*, 2005). MCTPs have been implicated in neuronal function and development in animal models (Lalani *et al.*, 2013; Qiu *et al.*, 2015; Genç *et al.*, 2017; Espino-Saldaña *et al.*, 2020). Humans have two homologues of MCTPs, MCTP1 and MCTP2, and they have been shown to play an important lipid droplet (LD) biogenesis (Joshi *et al.*, 2018, 2021). Live-imaging in human cells has shown that MCTPs localize at multiple ER-organelle contact sites, including ER-LD, ER-peroxisome, ER-mitochondria, and ER-endosome (Joshi *et al.*, 2021). Plant MCTP homologues MCTP3, 4, and 6 are shown to act as ER–PM contact sites at plasmodesmata (Brault *et al.*, 2019; Li *et al.*, 2024; Pérez-Sancho *et al.*, 2025).

In this report, using the proximity labeling assay, miniTurboID, we show that MCTP1 and MCTP2 localize to multiple ER-organelle contact sites. We confirm that MCTPs localize at ER-plasma MCS and promote them in C2 domain-dependent manner. We show that C2-domains bind to PI(4)P and PI(4,5)P_2_, which might be critical in localizing MCTPs at ER–PM contact sites. Furthermore, we demonstrate that MCTP1 and MCTP2 are important in maintaining PI(4)P homeostasis and cell migration.

## RESULTS AND DISCUSSION

### Proximity interactome analysis shows that MCTPs are localized at multiple ER-organelle contact sites

**FIGURE 1:**
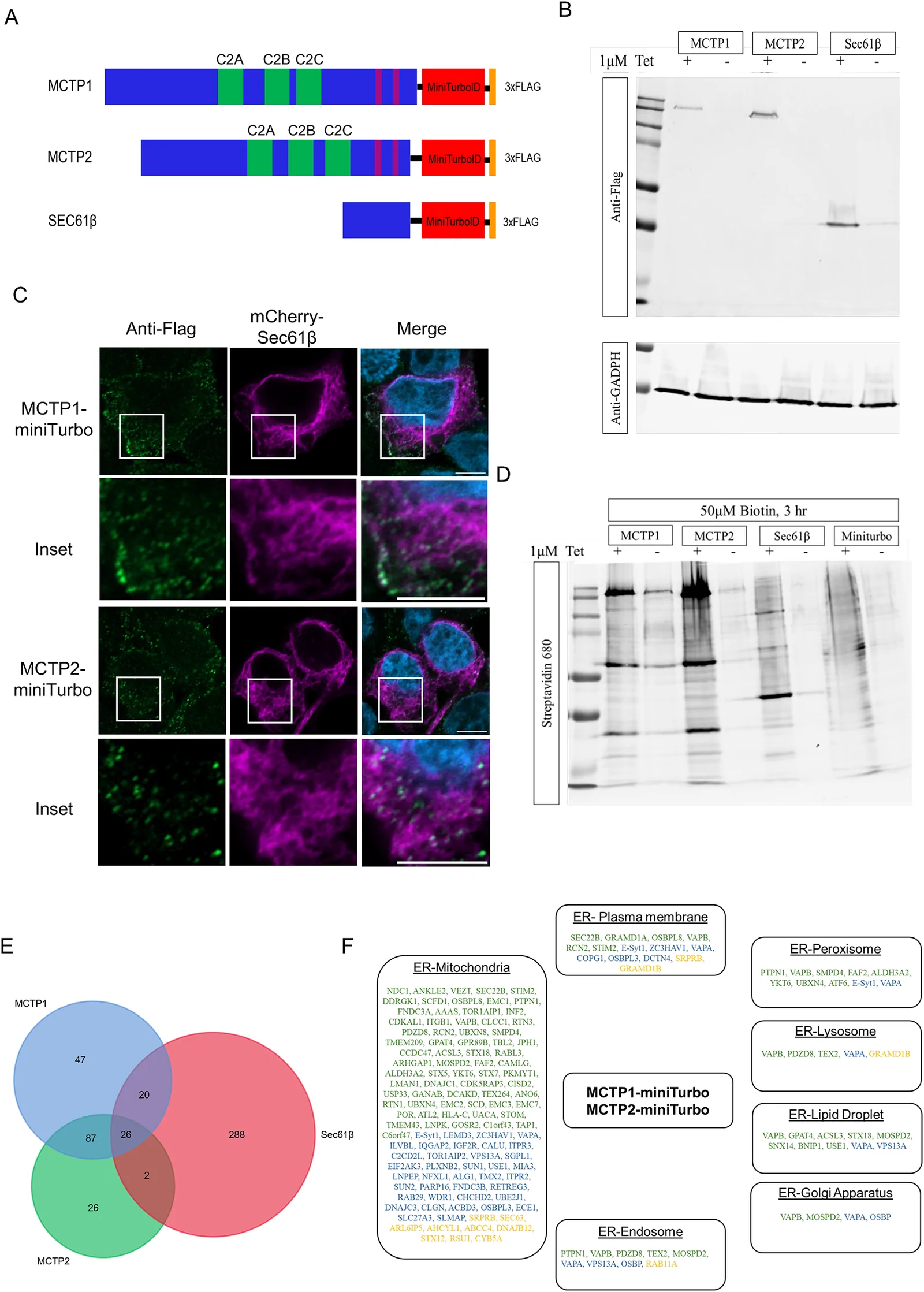
Proximity labeling approach shows MCTPs are at multiple ER-organelle contact sites. (A) Domain architecture of MCTP1-miniTurbo-3xFlag, MCTP2-miniTurbo-3xFlag, and SEC61β-miniTurbo-3xFlag constructs. C2 domains in green, and RHD in magenta. (B) Immunoblot of MCTP1-miniTurbo-3xFlag, MCTP2-miniTurbo-3xFlag, and Sec61b-miniTurbo-3xFlag expressed with 1 µM tetracycline induction. Anti-flag (1:2000) antibody was used to visualize the expression, and anti-GADPH antibody (1:5000) was used as a loading control. (C) Airyscan images of HEK293T stable lines expressing MCTP1-miniTurbo-3xFlag, MCTP2-miniTurbo-3xFlag, when induced with 1 µM Tetracycline. Cells were transfected with mcherry-SEC61β (ER marker), fixed, and stained for FLAG and stained with DAPI; scale bar, 5 µm. (D) Confirmation of biotinylated proteome with after tetracycline induction and incubation with 50 µM Biotin. Biotinylated proteomes visualized with fluorescently labeled streptavidin (1:2000). (E) MCTPs show unique high-confidence interactors (FDR< 0.02) compared with pan-ER protein SEC61β. (F) Known ER-organelle MCS proteins in MCTPs’ high-confidence interactors identified using MCSdB database. High-confidence interactors of both MCTP1-miniTurbo and MCTP2-miniTurbo are in green, only MCTP1-miniTurbo in blue, and only MCTP2-miniTurbo in yellow.

To determine the interactome of MCTPs, we performed a proximity-dependent biotinylation assay using TurboID (Kim *et al.*, 2014). We used the miniTurbo variant of BirA^*^ promiscuous biotin ligase (Branon *et al.*, 2018). We generated stable HEK293T cell lines that express MCTP1 and MCTP2 tagged with C-terminal miniTurbo-3xFlag under a tetracycline-inducible promoter using the Flp-In T-REx site-specific recombination system (O'Gorman *et al.*, 1991). A stable line expressing Sec61β tagged with C-terminal miniTurbo-3xFlag was generated as a pan-ER control for proximity labeling (Figure 1A). Expression of recombinant MCTPs and Sec61β tagged with miniTurbo-3xFlag was confirmed with Western blot (Figure 1B). Localization of the MCTPs in the ER membrane at the expression level where the MCTPs form punctate was assessed with immunofluorescence after tetracycline induction (Figure 1C). Biotinylation of prey proteins was assessed with a Western blot after tetracycline induction and biotin treatment with labeled streptavidin (Figure 1D).

The proximity labeling assay resulted in 180, 141, and 336 high-confidence interactors (<2% FDR) of MCTP1, MCTP2, and Sec61β, respectively (Supplemental Table S1). MCTP1 and MCTP2 shared 113/180 and 113/141 of high-confidence interactors, respectively. On the other hand, MCTP1 and MCTP2 shared only 46/180 and 28/141 with Sec61β, respectively (Figure 1E). The unique interactome obtained for MCTPs compared with the pan-ER marker, Sec61β, confirms that MCTPs are enriched in discrete ER subdomains (Joshi *et al.*, 2021). The high number of shared high-confidence interactors between the two MCTPs could be due to shared spatial localization of MCTPs and potentially redundant function.

We used MCSdb to determine whether the high-confidence interactors of MCTPs are known MCS proteins (Pan *et al.*, 2024). We identified many known MCS proteins in high-confidence interactors of both MCTP1 and MCTP2. We found high-confidence interactors identified at ER-LD contact sites, which is consistent with our previous findings (Joshi *et al.*, 2021) These data confirm that ER subdomains enriched with MCTPs are in close proximity with multiple organelles (Figure 1F).

### MCTPs localize at ER–PM contact sites

**FIGURE 2:**
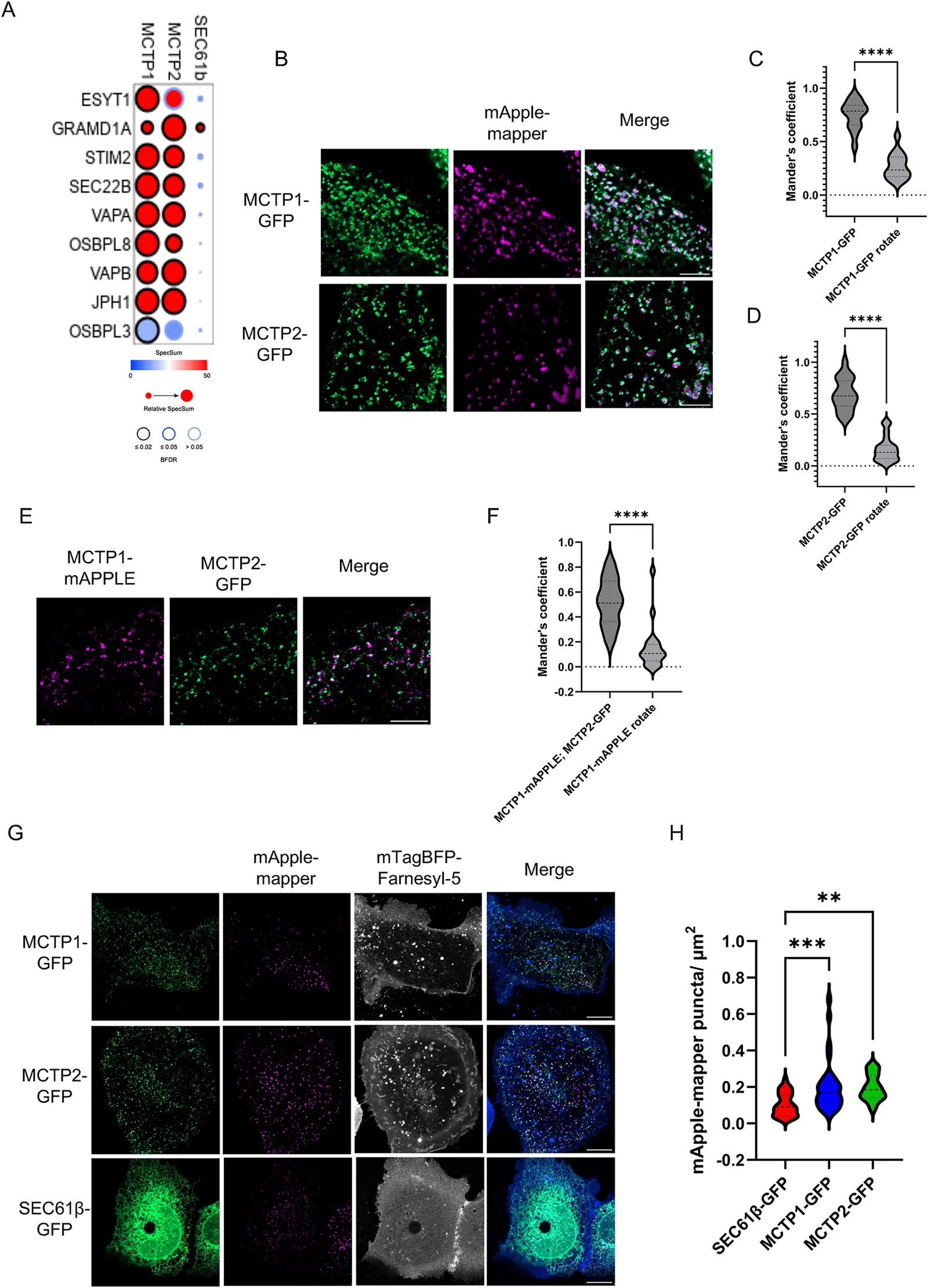
MCTPs are ER–PM contact site proteins. (A) Dot plot of known ER–PM MCS proteins from MCTP1/2-miniTurbo high-confidence interactors, displaying prey abundance across baits and prey confidence. Dot plot was generated by ProHit-viz web tool. (B) Airyscan images of U2OS cells expressing MCTP1-GFP or MCTP2-GFP and mApple-mapper; scale bar, 5 µm. (C) Quantification of MCTP1-GFP and mApple-mapper colocalization using Mander's colocalization coefficient. *n* = 15 cells from two independent experiments. ****p < 0.0001, Student *t* test. (D) Quantification of MCTP2-GFP and mApple-mapper colocalization using Mander's colocalization coefficient. *n* = 19 cells from two independent experiments. ****p < 0.0001, Student *t* test. E. Airyscan images of U2OS cells expressing MCTP1-mApple and MCTP2-GFP; scale bar, 5 µm. (F) Quantification of MCTP1-mApple and MCTP2-GFP colocalization using Mander's colocalization coefficient. *n* = 19 cells from two independent experiments. ****p < 0.0001, Student *t* test. (G) Airyscan images of U2OS cells expressing MCTP1-GFP, MCTP2-GFP, and SEC61β-GFP with mApple-mapper and mTagBFP-Farnesyl-5; scale bar, 5 µm. (H) Quantification of mApple-mapper puncta per µm^2^. SEC61β-GFP (*n* = 27), MCTP1-GFP (*n* = 30), and MCTP2-GFP (*n* = 30) cells from three independent experiments. ***p <0.001, one-way ANOVA with Tukey's test.

MCTP1 and MCTP2 show high-confidence interactions with known ER–PM contact site proteins such as ESYT1, STIM2, SEC22B, VAPA, OSBPL8, and JPH1 (Figure 2A). This suggests that MCTPs could be localized at ER–PM contact sites. To validate the localization of MCTPs at ER–PM contact sites, we expressed MCTP1 or MCTP2 tagged with green fluorescent protein (GFP) at the C-terminal with mApple-mapper, an ER–PM contact sites marker, in U2OS cells and imaged at the basal membrane using Airyscan (Chang *et al.*, 2013). Both MCTP1-GFP and MCTP2-GFP puncta colocalize with mApple-mapper (Figure 2B). Quantification of colocalization showed Mander's colocalization coefficient of 0.7503 and 0.6900, respectively. This colocalization was not random, as clockwise rotation of MCTP1-GFP or MCTP2-GFP reduced the Mander's colocalization coefficient to 0.2704 and 0.1627, respectively (Figure 2, C and D). This finding confirms the localization of human MCTPs at the ER–PM contact sites. These data agree with the previous report showing plant MCTP homologues at the ER–PM contact sites enriched in plasmodesmata (Brault *et al.*, 2019; Li *et al.*, 2024; Pérez-Sancho *et al.*, 2025).

To determine whether both MCTP1 and MCTP2 are localized at the same ER–PM contact sites, we co-expressed MCTP1-mApple and MCTP2-GFP and imaged the basal cell membrane. MCTP1 puncta colocalized with MCTP2, with Mander's colocalization coefficient of 0.5198 (Figure 2, E and F). Clockwise rotation of MCTP2-GPF reduced the Mander's colocalization coefficient of 0.1518, indicating that MCTP1 colocalization with MCTP2 is not random (Figure 2F). These data along with shared high-confidence interactors of MCTP1 and MCTP2 (Figure 1E), suggest that both MCTPs are enriched at the same subdomains of the ER.

### Overexpression of MCTPs increases ER–PM contact sites

Next, we checked whether MCTPs affect ER–PM contact sites. To do that, we overexpressed MCTP1-GFP, MCTP2-GFP, or GFP-SEC61β in U2OS cells expressing mApple-mapper and mTagBFP-Farnesyl-5 and imaged ER–PM contact sites at the basal cell membrane. Overexpression of GFP-SEC61β, MCTP1-GFP, and MCTP2-GFP showed 0.1035, 0.2032, and 0.1972 mApple-mapper puncta/µm^2^, respectively (Figure 2, G and H), indicating that overexpression of either MCTP1-GFP or MCTP2-GFP is sufficient to increase ER–PM contact sites. This finding suggests a potential role of MCTPs as a tether in promoting ER–PM contact sites.

Next, we checked whether loss of MCTPs alters ER–PM contact sites. We generated MCTP1 and MCTP2 knockout (KO) cell lines in the U2OS background using CRISPR-CAS9–mediated mutagenesis. We targeted exon 9 of MCTP1 and exon 10 of MCTP2 (Supplemental Figure S1, A and B) with gRNAs (Supplemental Table S2). After antibiotic selection and clonal expansion, clones with insertions or deletions were selected (Supplemental Figure S1, C and D) and sequenced to identify frame-shift mutants. We used the MCTP1 KO cell clone with 110 bp deletion and the MCTP2 KO cell clone with 17 bp for further studies (Supplemental Figure S1, E and F). We also confirmed the CRISPR KO by checking mRNA transcripts of MCTPs with reverse-transcriptase PCR (Supplemental Figure S1, G and H). We confirmed the MCTP2 KO by immunoblotting with MCTP2 antibody (Supplemental Figure S1, I and J). Also, we found that MCTP2 protein levels in MCTP1 KO cells were similar to WT (Supplemental Figure S1, I and J). Interestingly, MCTP1 and MCTP2 mRNA levels were downregulated in MCTP2 KO and MCTP1 KO cells, respectively (Supplemental Figure S1, K and L), indicating transcriptional regulation. MCTP1 KO and MCTP2 KO cells did not show any changes in ER–PM contact sites compared with WT (Supplemental Figure S2, A and B). This could be due to the potential compensatory role of other ER–PM contact site proteins in tethering ER–PM. It is not uncommon to not have any decrease in the number of ER–PM contact sites even with depletion of multiple tethering proteins. In yeast, ER–PM contact sites are greatly reduced in the six KO strain, but there are still some ER–PM contacts (Manford *et al.*, 2012). In mammals, triple KO of ESYT did not show any obvious defects in ER–PM contact (Sclip *et al.*, 2016; Tremblay and Moss, 2016). Our findings suggest that MCTPs regulate the ER–PM contact sites.

### C2 domains of MCTPs mediated PM contact

**FIGURE 3:**
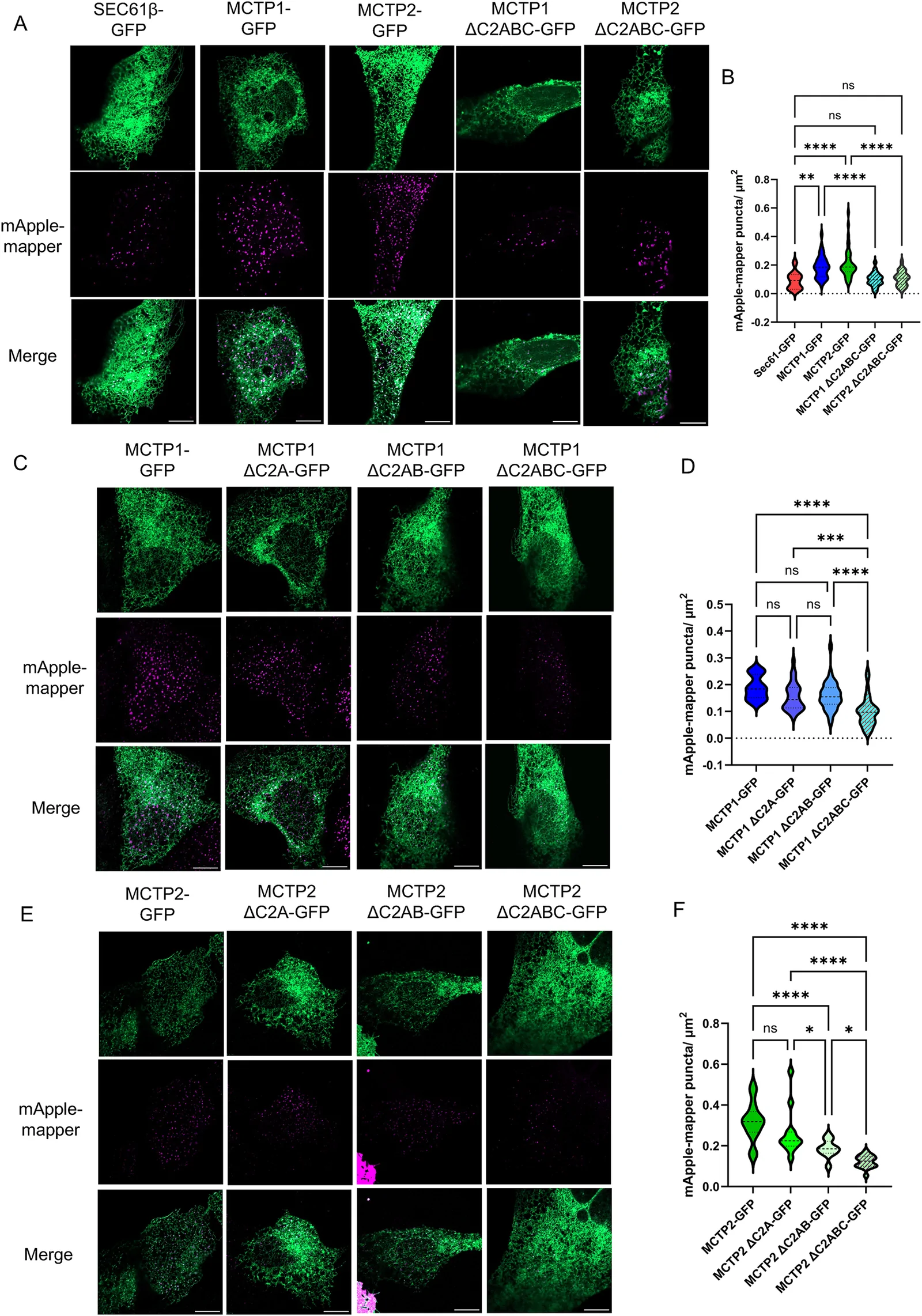
MCTPs’ localization at the ER–PM contact site requires C2 domains. (A) Airyscan images of U2OS cells expressing SEC61β-GFP, MCTP1-GFP, MCTP2-GFP, MCTP1-ΔC2ABC-GFP, MCTP2-ΔC2ABC-GFP, and mAPPLE-mapper; scale bar, 5 µm. (B) Quantification of mAPPLE-mapper puncta per µm^2^. SEC61β-GFP (*n* = 10), MCTP1-GFP (*n* = 36), MCTP2-GFP (*n* = 42), MCTP1-ΔC2ABC-GFP (*n* = 45), and MCTP2-ΔC2ABC-GFP (*n* = 44) cells from three independent experiments. ****p <0.0001, **p < 0.01, ns < 0.05, One-way ANOVA with Tukey's test. (C) Airyscan images of U2OS cells expressing MCTP1-GFP, MCTP1-ΔC2A-GFP, MCTP1-ΔC2AB-GFP, MCTP1-ΔC2ABC-GFP, and mAPPLE-mapper; scale bar, 5 µm. (D) Quantification of mAPPLE-mapper puncta per µm^2^. MCTP1-GFP (*n* = 14), MCTP1-ΔC2A-GFP (*n* = 32), MCTP1-ΔC2AB-GFP (*n* = 32), and MCTP1-ΔC2ABC-GFP (*n* = 32) cells from three independent experiments. ****p < 0.0001, ***p < 0.001, ns < 0.05, One-way ANOVA with Tukey's test. (E) Airyscan images of U2OS cells expressing MCTP2-GFP, MCTP2-ΔC2A-GFP, MCTP2-ΔC2AB-GFP, MCTP2-ΔC2ABC-GFP, and mAPPLE-mapper; scale bar, 5 µm. (F) Quantification of mAPPLE-mapper puncta per µm^2^. MCTP2-GFP (*n* = 14), MCTP2-ΔC2A-GFP (*n* = 12), MCTP2-ΔC2AB-GFP (*n* = 20), and MCTP2-ΔC2ABC-GFP (*n* = 18) cells from three independent experiments. ****p < 0.0001, *p < 0.05, ns < 0.05, One-way ANOVA with Tukey's test.

MCTP1 and MCTP2 contain three C2 domains, C2A, C2B, and C2C (Shin *et al.*, 2005). C2 domains are well-known lipid-binding domains with a wide range of lipid specificity and calcium dependency (Lemmon, 2008; Elíes *et al.*, 2019). In silico analysis of the MCTP C2 domains shows putative calcium and lipid binding pockets (Téllez-Arreola *et al.*, 2022). Many ER–PM tether proteins interact with PM through lipid-binding domains, including C2 domains in ESYT (Saheki *et al.*, 2016; Li *et al.*, 2021). To understand the role of MCTP C2 domains in ER–PM contact sites, we coexpressed truncated MCTPs that lack all three C2 domains (MCTP1 ΔC2ABC and MCTP2 ΔC2ABC) with mApple-mapper and mTagBFP-Farnesyl-5 in U2OS cells. We used GFP-tagged full-length MCTP1, MCTP2, and Sec61β as controls. Overexpression of truncated MCTP1 ΔC2ABC and MCTP2 ΔC2ABC did not increase ER–PM contact sites (Figure 3, A and B). The number of ER–PM contact sites for truncated MCTPs was comparable with overexpression of Sec61β (Figure 3, A and B). Thus, MCTP C2 domains are important in regulating the number of ER–PM contact sites.

To understand whether any individual C2 domains preferentially play a role in tethering ER–PM contact sites, we expressed GFP-tagged truncated MCTP1 and 2 that lack C2A (MCTP1/2 ΔC2A), C2A and C2B (MCTP1/2 ΔC2AB), and C2A, C2B, and C2C (MCTP1/2 ΔC2ABC) along with mApple-mapper and mTagBFP-Farnesyl-5. Overexpression of full-length MCTP1/2, MCTP1/2 ΔC2A, and MCTP1/2 ΔC2AB shows an increase in ER–PM contact sites compared with MCTP1 ΔC2ABC, which lacks all three C2 domains (Figure 3, C−F). These data suggest that the single C2 domain of MCTP1 is sufficient for regulating ER–PM contacts. Interestingly, in case of MCTP2, C2B and C2C show a synergistic role in regulating ER–PM contacts (Figure 3F).

MCTP RHD domains were shown to tubulate the ER (Joshi *et al.*, 2021). To study whether MCTP RHD regulates ER–PM contact site, we expressed MCTP2-GFP, the MCTP2 chimera that has RHD domain replaced with Sec61β tail anchor (MCTP2-Sec61β), or the MCTP2 truncation that lacks the C2 domains (MCTP2 ΔC2ABC) with mApple-mapper. Overexpression of both full-length MCTP2 and MCTP2-Sec61β chimera showed an increase in ER–PM MCS compared with MCTP2 ΔC2ABC (Supplemental Figure S2, C and D). Interestingly, we find that MCTP2-Sec61β-GFP shows stronger colocalization with mApple-mapper than MCTP2-GFP (Figure 2B; Supplemental Figure S2C), possibly because MCTPs diffuse more slowly than Sec61β. Moreover, MCTPs are restricted to regions with membrane curvature (Joshi *et al.*, 2021). This finding suggests that the ability of the MCTP2 RHD domain to tubulate ER is not essential for regulating ER–PM contact sites.

### MCTP C2 domains bind PI(4)P and PI(4,5)P_2_

**FIGURE 4:**
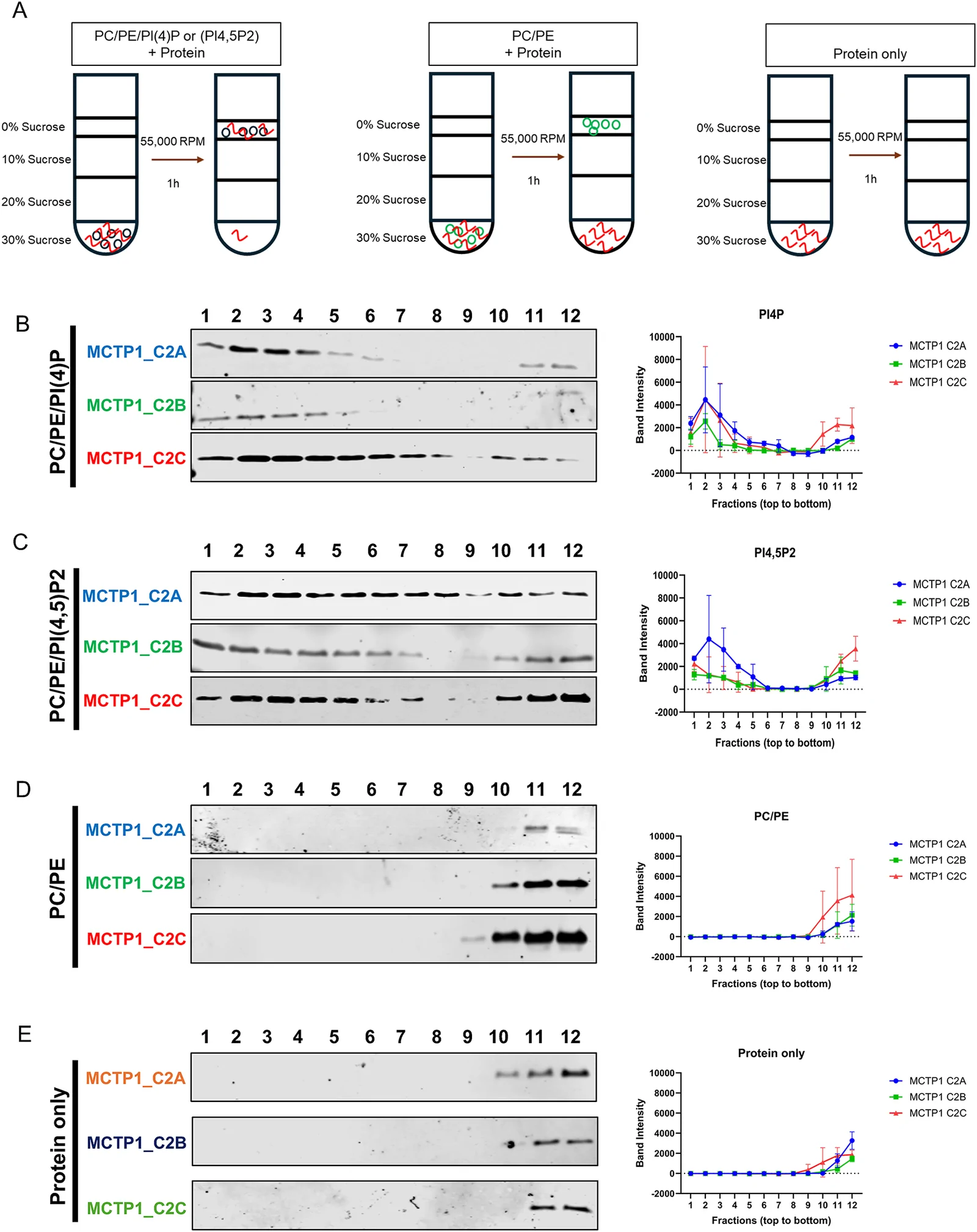
MCTP1 C2 domains bind to PI(4)P and PI(4,5)P_2_. (A) Diagram of liposome flotation assay by sucrose density gradient centrifugation. (B−E) Twelve fractions (1−12, top−bottom) were collected post gradient centrifugation, resolved on Western blot, and stained with anti-His antibody to evaluate the presence of C2 domains. Quantification of protein enrichment shown on the side of each gel.

C2 domains are known for their ability to bind lipids (Lemmon, 2008; Elíes *et al.*, 2019). ESYT, another C2 domain containing ER–PM tether, interacts with PM through its ability to bind PI(4,5)P_2_ via C2 domains. PI(4)P and PI(4,5)P_2_ are the major phosphoinositides in the PM, and other ER–PM tether proteins such as ESYT, OSBPL3/5/8, and Nir2/3 use the ability to bind these lipids to tether with the PM (Kim *et al.*, 2013; Chung *et al.*, 2015; Saheki *et al.*, 2016; Ghai *et al.*, 2017; Gulyás *et al.*, 2020). Previously, we showed that cell lysates expressing all three MCTP C2 domains bind to PIPs using a lipid overlay strip (Joshi *et al.*, 2021). To check the ability of individual C2 domains to bind to phospholipids, we expressed 6xHis-tagged individual C2 domains in *E. coli* and purified them with Ni-NTA affinity chromatography followed by size exclusion chromatography (SEC). We used the purified C2 domains from MCTP1 to perform the liposome flotation assay to determine whether they can bind to PI(4)P and PI(4,5)P_2_. To perform liposome flotation assay, we prepared liposomes comprising 75% PC/20% PE/ 5% PI(4)P and 75% PC/20% PE/ 5% PI(4,5)P_2_ to study MCTP C2 domain binding to PI(4)P and PI(4,5)P_2_. We incubated purified C2 domains with the liposomes and resolved them on a sucrose gradient to assess whether the proteins floated with liposomes. We used 80% PC/ 20%PE liposome and protein-only floatation as controls (Figure 4A).

All three C2 domains of MCTP1 bind liposomes containing PI(4)P (Figure 4B) and PI(4,5)P_2_ (Figure 4C) as they appear in the top fractions. On the other hand, only PC/PE containing liposomes (Figure 4D) or protein only (Figure 4E) controls did not show the presence of MCTP1 C2 domains in the top fractions. These data suggest that all three C2 domains of MCTP1 are able to bind PI(4)P and PI(4,5)P_2_, the major phosphoinositides enriched in PM. The binding of C2 domains to the two PIPs could be charge-dependent and nonspecific. Thus, our findings confirm the ability of MCTPs to regulate the number of ER–PM contact sites in C2 domain-dependent manner.

### MCTP KO cells exhibit increased PI(4)P but not PI(4,5)P_2_ levels at PM

**FIGURE 5:**
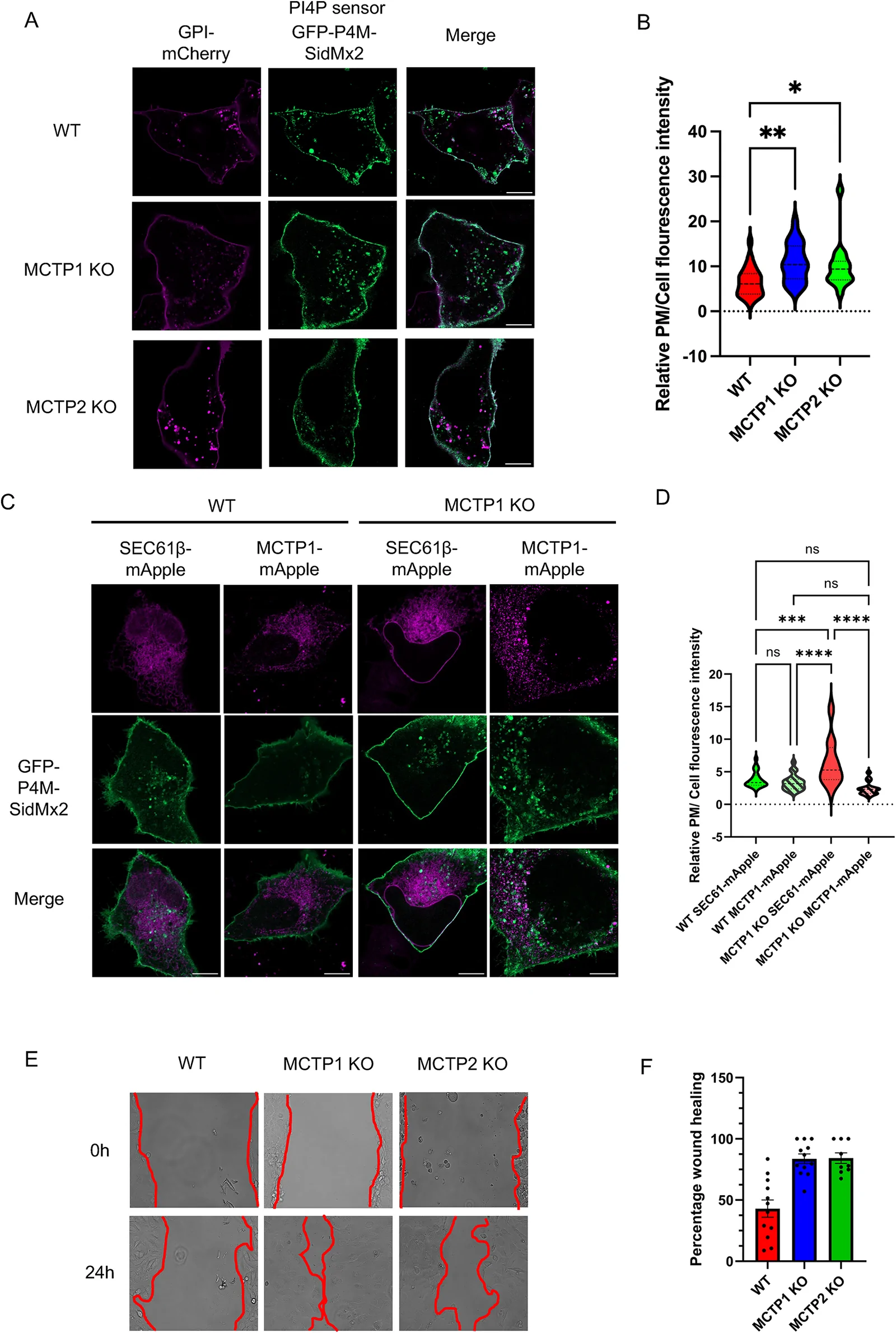
MCTP KO cells exhibit elevated PI(4)P level in PM and promote cell migration. (A) Airyscan images of WT and MCTP1 and MCTP2 KO cells expressing GFP-P4M-SidMx2, PI(4)P sensor, and GPI-mCherry, PM marker; scale bar, 5 µm. (B) Quantification of PI(4)P sensor intensity at PM relative to cytosol. WT (*n* = 25), MCTP1 KO (*n* = 24), and MCTP2 KO (*n* = 15) cells from two independent experiments. **p < 0.01, **p < 0.01, *p < 0.05, One-way ANOVA with Tukey's test. (C) Airyscan images of WT and MCTP1 KO cells expressing GFP-P4M-SidMx2, PI(4)P sensor, and MCTP1-mApple or SEC61β-mApple; scale bar, 5 µm. (D) Quantification of PI(4)P sensor intensity at PM relative to cytosol. WT expressing SEC61β-mApple (*n* = 21), WT expressing MCTP1-mApple (*n* = 20), MCTP1 KO expressing SEC61β-mApple (*n* = 20), and MCTP1 KO expressing MCTP1-mApple (*n* = 16) from three independent experiments. ****p < 0.0001, ***p < 0.001, ns < 0.05 One-way ANOVA with Tukey's test. (E) Brightfield images of WT, MCTP1, and 2 KO cells 0 and 24 h after wound-healing assay. (F) Quantification of wound healing as a ratio of healed length to initial wound length. WT (*n* = 12), MCTP1 KO (*n* = 12), and 2 KO (*n* = 9) from three independent experiments. ****p < 0.0001, One-way ANOVA with Tukey's test.

ER–PM MCS play a major role in the PI cycle, and ER–PM tether proteins perform lipid exchange as a part of PI cycle to maintain PM lipid homeostasis (Henne *et al.*, 2015; Epand, 2017; Li *et al.*, 2021). To understand whether MCTPs play any role in PM lipid homeostasis, we expressed GFP-P4M-SidMx2 and PH-PLCD-GFP as sensors to check PI(4)P and PI(4,5)P_2_, levels in the PM, respectively, with GPI-mCherry or mTagBFP-Farnesyl as the PM marker in WT U2OS, MCTP1 KO, and MCTP2 KO cells. Fluorescence intensity of PI(4)P and PI(4,5)P_2_ sensors at the PM was measured relative to cytosol fluorescence intensity (Chen *et al.*, 2017). MCTP1 and MCTP2 mutants showed elevated PI(4)P levels compared with WT cells (Figure 5, A and B). We did not see any significant change in PI(4,5)P_2_ level in MCTP mutants compared with wild-type (WT) (Supplemental Figure S3, A and B). Next, we coexpressed GFP-P4M-SidMx2, a PI(4)P sensor, with MCTP1-mApple or mApple-SEC61β in WT U2OS and MCTP1 KO cells. Overexpression of MCTP1-mApple in MCTP1 KO cells restored the PI(4)P levels to WT cells expressing mApple-SEC61β (Figure 5, C and D). Overexpression of MCTP1-mApple in MCTP1 KO cells showed the same level of PI(4)P as WT cells expressed with MCTP1-mApple or mApple-SEC61β (Figure 5, C and D). Whether tethering of ER and PM through MCTP C2 domains is required to regulate PM PI4P levels remains to be investigated. Taken together, these data show that loss of MCTPs alters PM PI(4)P level and indicates that MCTPs could play a role in PI cycle and PM lipid homeostasis.

### MCTP1 and MCTP2 KO cells show increased cell migration

ER–PM contact site gradient along the leading to the lagging edge of cells is important in cell migration (Gong *et al.*, 2024). A major ER–PM tether protein, VAPA, has been shown to play a critical role in cell migration and PI(4)P homeostasis at the PM (Siegfried *et al.*, 2024). As our data show that MCTPs are ER–PM contact site tethers and play a role in PI(4)P homeostasis at the PM, we checked whether MCTPs affect cell migration by performing a wound-healing assay. We cultured U2OS WT, MCTP1 KO, and MCTP2 KO cells in 12-well plates until 100% confluence and made a scratch with a P200 tip. We measure the wound distance at 0 and 24 h after scratching. MCTP1 or MCTP2 KO cells had more healed surfaces than WT controls (Figure 5E). WT, MCTP1 KO, and MCTP2 KO cells showed 43, 84, and 84% of wound healing, respectively (Figure 5F). Our data show that cells devoid of MCTPs exhibit increased cell migration compared with WT cells. Next, we checked whether C2 domains play a role in regulating cell migration. The full-length MCTP1-GFP and MCTP2-GFP were able to decrease cell migration upon overexpression in MCTP1 KO and MCTP2 KO cells, respectively (Supplemental Figure S3C). Interestingly, cells overexpressing MCTP1 ΔC2ABC- GFP increased cell migration as compared with cells overexpressing full-length MCTP1-GFP, indicating that MCTP1 C2 domain-dependent ER–PM tethering plays a role in cell migration. However, overexpression of MCTP2 ΔC2ABC-GFP reduced cell migration more than overexpression of full-length MCTP2-GFP in MCTP2 KO cells (Supplemental Figure S3C). Our findings suggest that ER–PM tethering by MCTP1 but not MCTP2 might regulate cell migration in C2 domain-dependent manner.

### MCTP1 and MCTP2 promote ER–PM contact sites in C2 domain-dependent manner

Taken together, we show that MCTP1 and MCTP2 are at multiple ER-organelle contact sites using biotinylation-based proximity labeling and live cell imaging. We show the localization of MCTPs at ER–PM MCS, and the overexpression of MCTPs increases ER–PM contact sites in C2 domain-dependent manner, confirming human MCTPs as ER–PM tethers. We also showed that C2 domains play a critical role in binding to PI(4)P and PI(4,5)P_2_, possibly facilitating the tethering function. MCTP KO cells showed elevated PM PI(4)P levels and increased cell migration. Elevation of PM PI(4)P indicates a potential role of MCTPs in the PI cycle. MCTPs could play a role in recruiting or assisting the OSBPL3 or 8 function, which are known to exchange PI(4)P from PM to ER (Ghai *et al.*, 2017; Gulyás *et al.*, 2020). Another potential reason for elevated PI(4)P could be changes in VAP-mediated recruitment of Sac1, a PI(4)P phosphatase. Being high-confidence interactors of MCTPs, OSBPL3, OSBPL8, and VAP proteins are potential prime targets for a mechanistic study to understand elevated PI(4)P levels in MCTP KOs. The ER–PM tether VAPA alters migration by anchoring ER–PM contact sites to focal adhesions and affects their dynamics (Siegfried *et al.*, 2024). ER–PM MCS protein OSBPL3, which exchanges PI(4)P, has been shown to regulate R-Ras signaling and cell migration (Weber-Boyvat *et al.*, 2015). Taken together, the migration defect observed in the MCTP KO could be related to ER–PM contact sites and PI(4)P homeostasis.

## MATERIALS AND METHODS

### Cell culture and reagents

U2OS and HEK293T cells were maintained at 37°C with 5% CO_2_ in DMEM (CORNING, Manassas, VA) media supplemented with 10% FBS, 2 mM Glutamine (Life Technologies, Grand Island, NY), and 1x penicillin/streptomycin (Life Technologies, Grand Island, NY).

### Plasmids

Plasmids and primers used in this study are listed in Supplemental Table S2. GFP-tagged MCTPs were generated by cloning the PCR amplified DNA sequence of full-length MCTP1 and MCTP2 into the pcDNA3-GFP plasmid using T4 ligase. Both plasmid and PCR products were digested with KpnI and NotI for MCTP1 and BamHI and NotI for MCTP2 cloning. mApple-tagged MCTP1 was generated by swapping GFP with PCR-amplified mApple after excising GFP with XhoI and AgeI digestion. GFP-tagged C2 domain-truncated MCTPs were generated by cloning PCR amplified DNA sequence of MCTP1 ΔC2A (450-999), MCTP1 ΔC2AB (607-999), MCTP2 ΔC2A (341-878), and MCTP2 ΔC2AB (490-878) into BamHI and NotI digested pcDNA3-GFP vector using HiFi cloning (NEB BioLabs, Ipswich, MA). For the expression of individual C2 domains, DNA sequence corresponding to MCTP1 C2A (243-391), C2B (450-598), and C2C (607-755) and MCTP2 C2A (182-314), C2B (341-480), and C2C (490-638) was PCR amplified and cloned into BamHI digested PET15b vector using HiFi cloning (NEB BioLabs, Ipswich, MA). mApple-mapper was generated by cloning PCR-amplified mApple into AgeI and SpeI-digested GFP-mapper to excise GFP using HiFi cloning (NEB Biolabs, Ipswich, MA).

### Lipofectamine transfection

U2OS cells were cultured in 10 µg/ ml fibronectin (Millipore, Burlington, MA) coated 8-well chambered cover glass (Cellvis, Mountainview, CA). Each well was seeded with 15,000 cells. Cells were transfected with liposomes assembled with Lipofectamine 2000 (Invitrogen, Carlsbad, CA) and 50 to 400 ng/well of plasmids for 6 h. Lipofectamine transfection was performed in OptiMEM media (CORNING, Manassas, VA). OptiMEM was replaced after 6 h with imaging media composed of phenol red-free DMEM (CORNING, Manassas, VA) supplemented with 10% FBS, 2 mM Glutamine, and 1x penicillin/streptomycin.

### Image acquisition

Images were acquired with a Zeiss800/Airyscan laser scanning confocal microscope (Zeiss, Germany). Airyscan images were acquired using a 63X/1.4 NA objective lens at 37°C with 5% CO_2_. Images were processed with Airyscan processing with Zeiss ZEN software packages. Image analyses were done with Fiji ImageJ (version 2.14.0). Images shown are from a single focal plane.

### Establishment of MCTP-miniturboID stable line

MCTP1, MCTP2, and Sec61β full-length coding sequences were cloned into pcDNA_FRT/TO_[MCS]-miniTurboID-3x FLAG plasmid that would express genes under a tetracycline-inducible promoter (Yan *et al.*, 2022). HEK293T-REx Flp-In cells were transfected with 1 µg MCTP or Sec61β Miniturbo construct plasmid and 2 µg of pOG44 using Lipofectamine 2000 (Invitrogen, Carlsbad, CA). Cells were selected with 200 µg/ ml Hygromycin B (Invitrogen, Carlsbad, CA) for stable integration. MCTP1, 2, and SEC61β expression were induced with 1 µM tetracycline (Life Technologies, Grand Island, NY) overnight at 37°C. Postinduction cells were incubated with 50 µM Biotin for 3 h for biotinylation. Cells were scraped with ice-cold PBS and pelleted by centrifugation. Sample preparation, mass spectrometry, and data processing were carried out as previously reported (Yan *et al.*, 2022).

### CRISPR-CAS9–mediated KO cell generation

Both MCTP1 exon 9 and MCTP2 exon 10 were targeted with two gRNA each, designed using CHOPCHOP Version 3 (Labun *et al.*, 2019; 2021). gRNAs were cloned into pSpCas9(BB)-2A-Puro (Addgene: 62988) according to a previously reported protocol (Ran *et al.*, 2013). One million cells were transfected with 1 µg of gRNA plasmids with Amaxa Cell Line Nucleofector Kit V (Lonza, Greenwood, SC) using X-001 program in Amaxa Nucleofector IIb. Transfected cells were seeded in 6-well plates and selected for puromycin (Life Technologies, Grand Island, NY) until resistant clones arose. Puromycin-resistant colonies were trypsinized and seeded in 96-well plates at 0.5 cells/well. Further clonal expansion was carried out subsequently in 24-well and 6-well plates. Genomic DNA was extracted from expanded clones using the Quick-DNA miniprep plus kit (ZymoResearch, Irvine, CA). Genomic DNA region targeted with gRNA amplified with MCTP1 forward-TGTACTTCTGATGCCCAAGC, MCTP1 reverse-TCAATTTTCATTTGCCAAAAGTT, MCTP2 forward-AGCAATGAATGACATTTTAAGAAA, MCTP2 reverse-GCCCATCCTGTCAGAGAAGT with PCR conditions given below. Initial denaturation: 95°C for 2 min, denaturation: 95°C for 20 s, annealing: 56°C for 20 s, extension: 72°C for 30 s for 35 cycles and final extension: 72°C for 5 min. PCR products were resolved on 15% polyacrylamide gel (VanLeuven *et al.*, 2018) to identify KO cell clones. PCR product clones showing deletion were cloned into a TA cloning vector according to the manufacturer's protocol (Invitrogen, Carlsbad, CA) and sequenced (Eurofins Sequencing, Louisville, KY).

### Western blot analysis

MCTP2 protein level was checked by Western blot analysis using MCTP2 polyclonal antibody (17578-1-AP, Proteintech), primary antibody used at 1:1000 dilution and detected with donkey anti-rabbit IgG (926-32213, LI-COR Biotech) used at 1:10,000 dilution. The quantification of the bands was performed using GelQuant software (version 1.8.2).

### RNA extraction and reverse-transcriptase PCR

Total RNA was extracted from WT, MCTP1 KO, and MCTP2 KO cells using the Quick-RNA miniprep kit (Zymo Research, Irvine, CA). First-strand cDNA was synthesized with 1 µg total RNA using the LunaScript RT Supermix kit (NEB Biolabs, Ipswich, MA). PCR product targeting MCTP1/2 C2B region amplified with MCTP1 forward-TTTCAGACCCAAAGTTTACGCCT, MCTP1 reverse-GTTGACAGACAGGTCAGAGATG, MCTP2 forward-CTACGGCTCTCTGAGTCC, MCTP2 reverse-GACACACAGATCAGAGACGG, with the following PCR conditions. Initial denaturation: 95°C for 2 min, denaturation: 95°C for 20 s, annealing: 56°C for 20 s, extension: 72°C for 30 s for 35 cycles, and final extension: 72°C for 5 min. PCR products were resolved 1.5% agarose gel.

### Quantitative RT-PCR

Amplification reactions were performed using first-strand cDNA synthesized from WT, MCTP1 KO, and MCTP2 KO cells. Reactions were set up in triplicate using PowerTrack SYBR Green Master Mix (Applied Biosystems, Vilnius, Lithuania) and run on a QuantStudio 5 Real-Time PCR System (Applied Biosystems) according to the manufacturer's instructions. The following primers were used: RPS16 forward-AAACGCGGCAATGGTCTCATCAAG, RPS16 reverse-TGGAGATGGACTGACGGATAGCAT, MCTP1 forward-GGACAAAGATGCTGGGAAAAGGG, MCTP1 reverse-CTGATGCTGTCAGAGTGACCAG, MCTP2 forward-CCTTAGCAGACCTCAGCGAAAG, MCTP2 reverse-CTCTTCCCTGAGAAATCTGCCG. Average cycle threshold (*C*_t_) values from three independent biological replicates were calculated and normalized to the housekeeping gene RPS16 using the formula 2^(*C*_t__control − *C*_t__target). Statistical comparisons between two groups were performed using Student's *t* test, with *P* < 0.05 considered statistically significant.

### MCTP C2 domain expression and purification

C2 domains of MCTPs were cloned into the PET15b vector and transformed into *E. coli* T7 Express *lys*Y (NEB Biolabs, Ipswich, MA) strain. Single colonies of the bacterial strain harboring C2 domains were cultured overnight in 25 ml of LB medium with ampicillin (100 µg/ml) and chloramphenicol (25 µg/ml) at 37°C. Overnight culture was added to 1 l fresh LB medium until OD600 is between 0.4 and 0.6. Then, protein expression was induced with 1 mM IPTG at 30°C for 3 h. Cells were harvested and lysed in 20 mM HEPES with 50 mM NaCl with French press at 25,000 psi. C2 domains were purified by Ni-NTA affinity chromatography followed by SEC.

### Liposome flotation assay

Liposome was made with 80% PC and 20% PE or 75%, 20% PE, and 5% PI(4)P or PI(4,5)P_2_. Dried lipids were hydrated in 20 mM HEPES with 50 mM NaCl and subjected to ten freeze-thaw cycles. Then, dissolved lipids were passed through a 100 nm polycarbonate filter membrane with an extruder 31 times. A total of 2 mM liposomes mixed with 4 µM purified protein in 60 µl and incubated at room temperature for 1 h. After incubation, the reaction was mixed with an equal volume of 60% sucrose and placed at the bottom of the centrifugation tube. Then, 240 µl of 20% sucrose layer, 240 µl of 10% sucrose layer, and 120 µl of 0% sucrose layer were added. The tubes were centrifuged in an SW 55 Ti rotor (Beckman Coulter, Brea, CA) at 55,000 RPM for 1 h at 4°C. After centrifugation, 12 fractions of 60 ml were collected and visualized in a western blot after anti–6X-His antibody staining using a Li-COR Odyssey scanner (Li-COR Biosciences, Lincoln, NE).

### Wound-healing assay

U2OS WT, MCTP1 KO, and MCTP2 KO cells were grown in 12-well plates until 100% confluence. Then, a scratch was made with a P200 tip, and the media were switched from regular growing media to DMEM media supplemented with 1% FBS, 2 mM Glutamine, and 1x penicillin/streptomycin. The mean wound distance was measured at three different points per image at 0 and 24 h after scratching.

### Statistical analysis

All the statistical analyses were carried out by either Student's *t* test or one-way ANOVA with Tukey's test using GraphPad Prism version 9.5.1.

## Supporting information









## Abbreviations used:

ER: endoplasmic reticulum
KO: knockout
MCS: membrane contact sites
PI(4)P: phosphatidylinositol 4-phosphate
PI(4,5)P_2_: phosphatidylinositol 4,5-bisphosphate
PM: plasma membrane
WT: wild-type.
