# Supplementary material for: MCTP1 and MCTP2 promote ER–PM contact sites and regulate PI4P homeostasis and cell migration
Source: Mol Biol Cell. 2026 Apr 6;37(5):br16. doi: 10.1091/mbc.E25-09-0445 (PMC13537348; doi:10.1091/mbc.E25-09-0445)
Supplement: Supplementary file 1 [file mbc-37-br16-s001.pdf]

Supplemental Materials

Molecular Biology of the Cell

Amirthagunanathan *et al*.

Figure S1

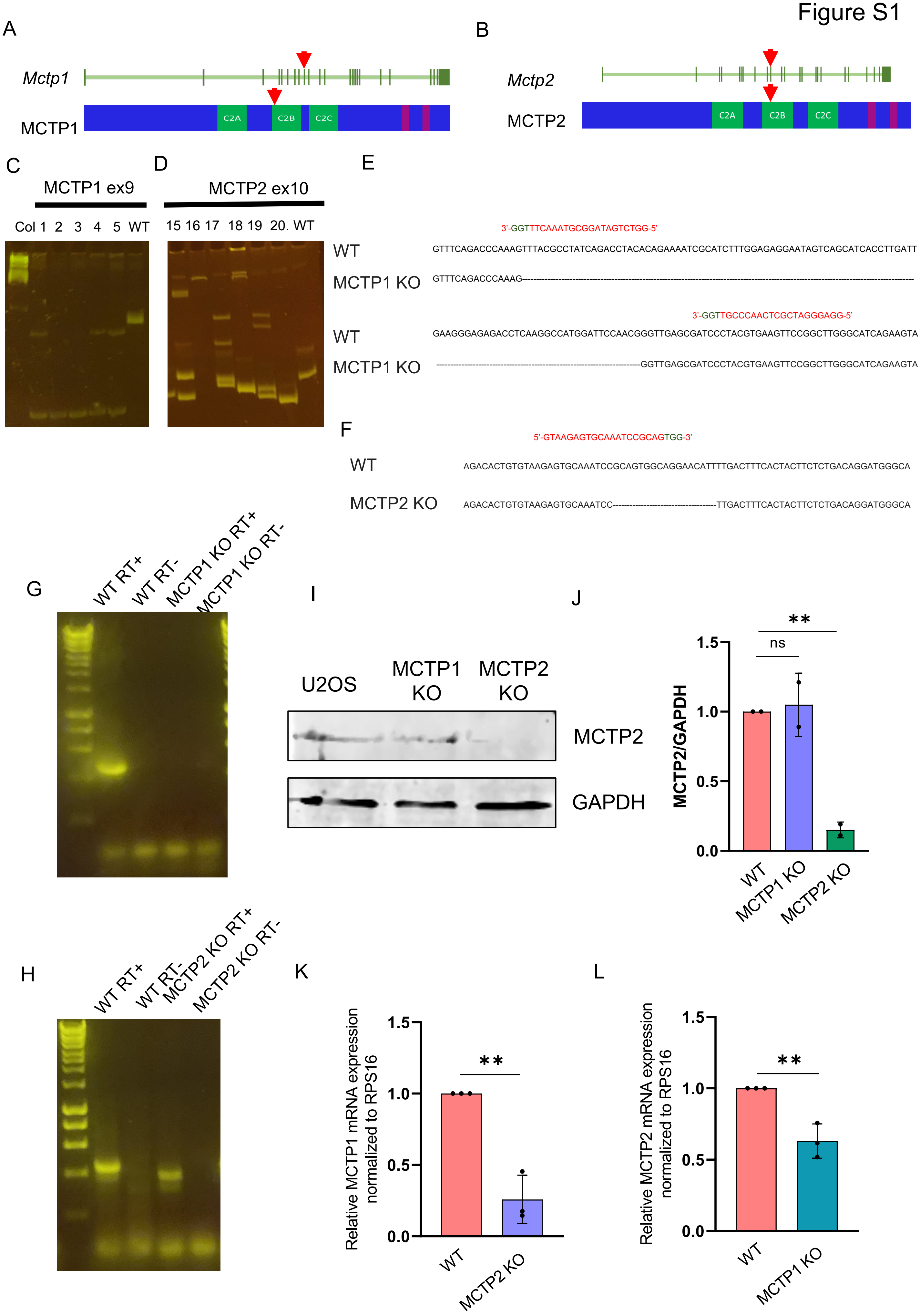
A
Mctp1
MCTP1
C2A
C2B
C2C
B
Mctp2
MCTP2
C2A
C2B
C2C
C
MCTP1 ex9
Col 1 2 3 4 5 WT
D
MCTP2 ex10
15 16 17 18 19 20. WT
E
3'-GGTTTCAAATGCGGATAGTCTGG-5'
WT
GTTTCAGACCCAAAGTTTACGCCTATCAGACCTACACAGAAAATCGCATCTTTGGAGAGGAATAGTCAGCATCACCTTGATT
MCTP1 KO
GTTTCAGACCCAAAG---
3'-GGTTGCCCAACTCGCTAGGGAGG-5'
WT
GAAGGGAGAGACCTCAAGGCCATGGATTCCAACGGGTTGAGCGATCCCTACGTGAAGTTCCGGCTTGGGCATCAGAAGTA
MCTP1 KO
---GGTTGAGCGATCCCTACGTGAAGTTCCGGCTTGGGCATCAGAAGTA
F
5'-GTAAGAGTGCAAATCCGCAGTGG-3'
WT
AGACACTGTGTAAGAGTGCAAATCCGCAGTGGCAGGAACATTTTGACTTTCACTACTTCTCTGACAGGATGGGCA
MCTP2 KO
AGACACTGTGTAAGAGTGCAAATCC-----------------------------TTGACTTTCACTACTTCTCTGACAGGATGGGCA
G
WT RT+
WT RT-
MCTP1 KO RT+
MCTP1 KO RT-
I
U2OS
MCTP1 KO
MCTP2 KO
MCTP2
GAPDH
J
MCTP2/GAPDH
1.5
1.0
0.5
0.0
ns
**
WT
MCTP1 KO
MCTP2 KO
H
WT RT+
WT RT-
MCTP2 KO RT+
MCTP2 KO RT-
K
Relative MCTP1 mRNA expression normalized to RPS16
1.5
1.0
0.5
0.0
**
WT
MCTP2 KO
L
Relative MCTP2 mRNA expression normalized to RPS16
1.5
1.0
0.5
0.0
**
WT
MCTP1 KO

Figure S2

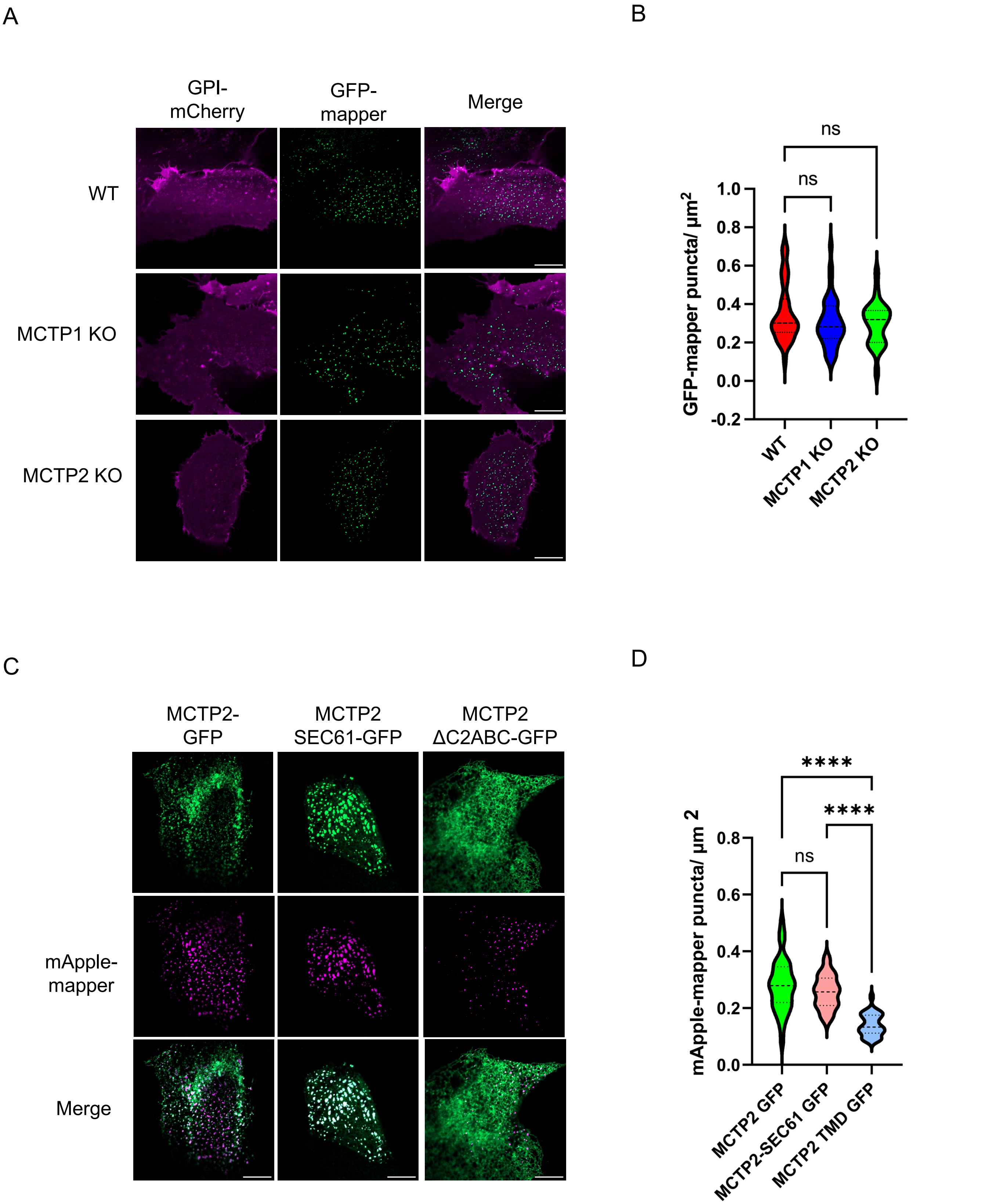
A
GPI-mCherry
GFP-mapper
Merge
WT
MCTP1 KO
MCTP2 KO
B
GFP-mapper puncta/ µm²
1.0
0.8
0.6
0.4
0.2
0.0
-0.2
ns
ns
WT
MCTP1 KO
MCTP2 KO
C
MCTP2-GFP
MCTP2 SEC61-GFP
MCTP2 ΔC2ABC-GFP
mApple-mapper
Merge
D
mApple-mapper puncta/ µm 2
0.8
0.6
0.4
0.2
0.0
****
****
ns
MCTP2 GFP
MCTP2-SEC61 GFP
MCTP2 TMD GFP

Figure S3

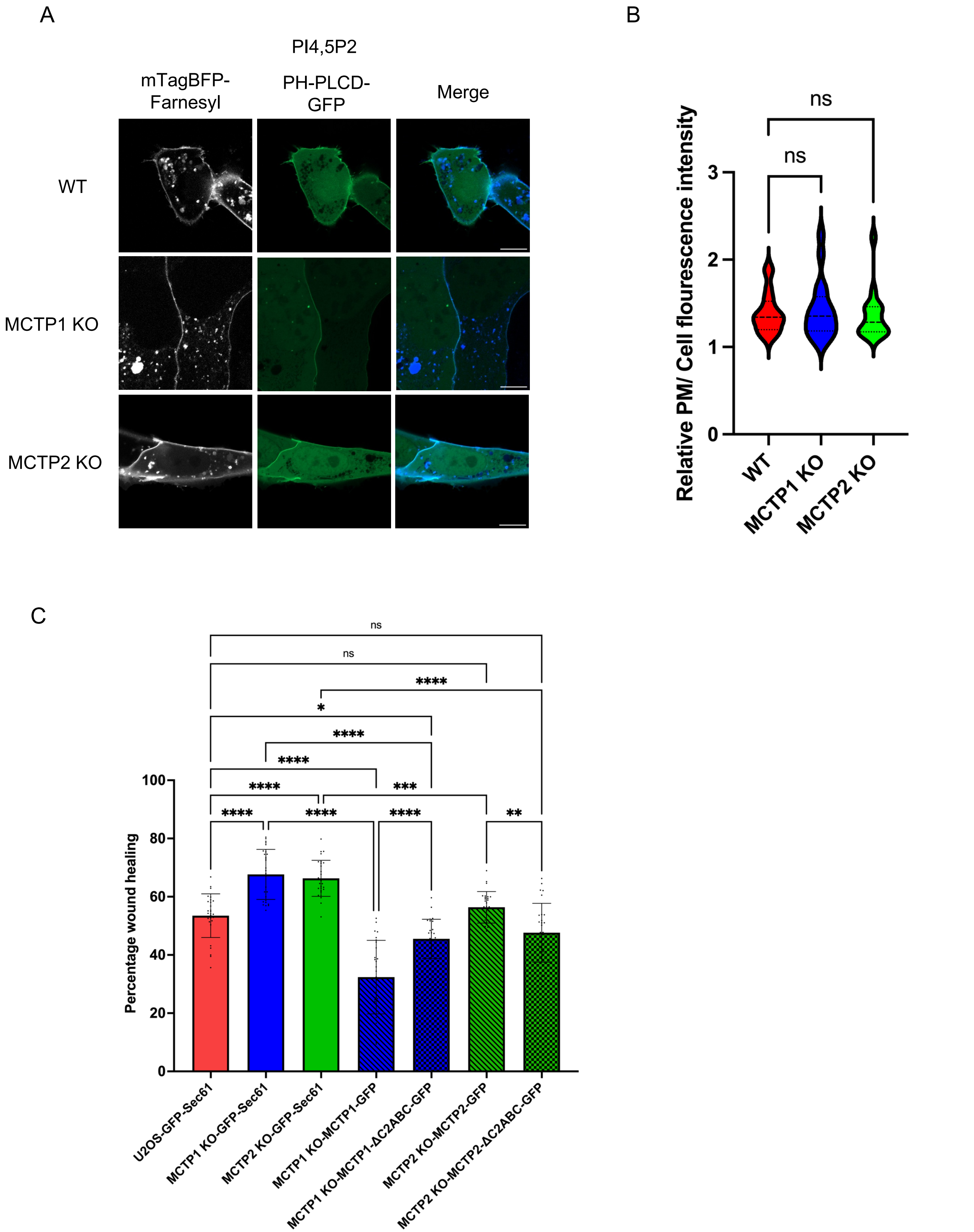
A
PI4,5P2
mTagBFP-Farnesyl
PH-PLCD-GFP
Merge
WT
MCTP1 KO
MCTP2 KO
B
ns
ns
Relative PM/ Cell flourescence intensity
3
2
1
0
WT
MCTP1 KO
MCTP2 KO
C
ns
ns
****
*
****
****
****
***
****
****
****
**
100
80
60
40
20
0
Percentage wound healing
U2OS-GFP-Sec61
MCTP1 KO-GFP-Sec61
MCTP2 KO-GFP-Sec61
MCTP1 KO-MCTP1-GFP
MCTP1 KO-MCTP1-ΔC2ABC-GFP
MCTP2 KO-MCTP2-GFP
MCTP2 KO-MCTP2-ΔC2ABC-GFP

Figure S1. Generation of MCTP1 and MCTP2 KO cells.

A. Schematic representation of gRNA target site of MCTP1 for CRISPR-CAS9 mediated mutagenesis. Two gRNAs targeting exon9 were used for mutagenesis.
B. Schematic representation of the gRNA target site of MCTP2 for CRISPR-CAS9-mediated mutagenesis. A gRNA targeting exon 10 was used for mutagenesis.
C. Screening for MCTP1 KO clones. PCR amplification was carried out with primers adjoining the gRNA target site and resolved on 15% PAGE gel to identify INDEL mutations.
D. Screening for MCTP2 KO clones. PCR amplification was performed with primers adjoining the gRNA target site and resolved on 15% PAGE gel to identify INDEL mutations.
E. Schematic representation of 101 bp deletion in MCTP1 KO cell.
F. Schematic representation of 17 bp deletion in MCTP2 KO cell.
G. Confirmation of MCTP1 mutation in MCTP1 mRNA. First-strand cDNA was synthesized from total RNA extracted from MCTP1 KO cells, and the coding region of MCTP1 C2B was amplified and resolved on a 1.5 % agarose gel to confirm the deletion in mRNA.
H. Confirmation of MCTP2 mutation in MCTP2 mRNA. First-strand cDNA was synthesized from total RNA extracted from MCTP2 KO cells, and the coding region of MCTP2 C2B was amplified and resolved on a 1.5 % agarose gel to confirm the deletion in mRNA.
I. Immunoblot analysis of cell lysates from WT U2OS, MCTP1 KO, and MCTP2 KO cells. Anti-MCTP2 antibody was used to determine the MCTP2 protein levels whereas anti-GAPDH antibody was used to detect GAPDH levels as a control.
J. Statistical comparisons between two groups were performed using Student’s t-test, with $P < 0.05$ considered statistically significant.

K and L. qRT-PCR analysis of MCTP1 mRNA levels in wild-type and MCTP2 KO cells, and MCTP2 mRNA level in wild-type and MCTP1 KO cells. Statistical comparisons between two groups were performed using Student's t-test, with $P < 0.05$ considered statistically significant.

Figure S2. Overexpression of MCTP constructs with altered TMDs does not increase ER-PM contact sites.

A. Airyscan images of WT U2OS, MCTP1 KO and MCTP2 KO cells expressing GFP-mapper and GPI-mCherry. Scale bar, 5µm.
B. Quantification of GFP-mapper puncta per μm^2. WT (*n* = 37), MCTP1 KO (*n* =40), and MCTP2 KO (*n* = 23) cells from three independent experiments., ns $p<0.05$, One-way ANOVA with Tukey's test.
C. Airyscan images of U2OS cells expressing MCTP2-GFP, MCTP2-SEC61-GFP, and MCTP2 ΔC2ABC-GFP and mAPPLE-mapper. Scale bar, 5µm.
D. Quantification of mAPPLE-mapper puncta per μm^2. MCTP2-GFP (*n* =32 cells), MCTP2-SEC61-GFP (*n* =30 cells), and MCTP2 ΔC2ABC-GFP (*n* =30 cells) from three independent experiments., ****$p<0.001$, One-way ANOVA with Tukey's test.

Figure S3. MCTP1 and MCTP2 KO does not affect PI(4,5)P_2 level in the PM.

A. Airyscan images of WT U2OS, MCTP1 KO and MCTP2 KO cells expressing PH-PLCD-GFP, a PI(4,5P)2 sensor, and mTagBFP-Farnesyl-5, PM marker. Scale bar, 5µm.
B. Quantification of PI(4,5)P_2 sensor intensity at PM relative to cytosol. WT (*n* = 18), MCTP1 KO (*n* = 17), and MCTP2 KO (*n* = 16) cells from three independent experiments. One-way ANOVA with Tukey's test.

C. Quantification of wound healing as a ratio of healed length to initial wound length. WT U2OS + GFP-Sec61β, MCTP1 KO + GFP-Sec61β, and MCTP2 KO + GFP-Sec61β, MCTP1 KO + MCTP1-GFP, MCTP1 KO + MCTP1- ΔC2ABC-GFP, MCTP2 KO + MCTP2-GFP, MCTP1 KO + MCTP1- ΔC2ABC-GFP from three independent experiments. *n*= 28, ****p<0.0001, One-way ANOVA with Tukey's test.
